# Comment on “Is the experience of chronic pain different in frail older adults? A cross-sectional exploratory study”

**DOI:** 10.1055/s-0046-1818605

**Published:** 2026-04-07

**Authors:** Shyam Sundar Sah, Abhishek Kumbhalwar

**Affiliations:** 1 Dr. D. Y. Patil Medical College Hospital and Research Centre, Dr. D. Y. Patil Vidyapeeth (Deemed-to-be-University), Pimpri, Pune, 411018, Maharashtra, India; 2 Dr. D. Y. Patil Dental College and Hospital, Dr. D. Y. Patil Vidyapeeth (Deemed-to-be-University), Pimpri, Pune, 411018, Maharashtra, India

Dear Editor,

We read with great interest the exploratory study by Cirino et al., 1 which offers a comprehensive analysis of chronic pain characteristics in older adults, stratified by frailty status. The integration of neuropathic pain, pain-induced depression, and multidimensional pain scales, alongside robust physical and psychosocial profiling, marks a valuable contribution to geriatric neurology and pain management.

However, cross-sectional comparisons may blur the underlying etiological distinctions regarding frailty and pain phenotypes. Frailty was operationalized using the Fried criteria, which emphasize physical components such as grip strength and gait speed. While this enables a standardized classification, it risks tautological inflation of the associations between pain and frailty, particularly when both constructs share physical-performance markers. 2 Clinically, this is important because the overlap may reflect diagnostic circularity rather than true mechanistic synergy. Alternative models, such as the deficit accumulation index, may better distinguish whether chronic pain drives frailty or if both arise from a common geroscience pathway.

A second point concerns the interpretation of neuropathic pain prevalence. The use of the Douleur Neuropathique en 4 Questions Questionnaire is appropriate for screening; however, the study treated scores ≥ 4 as a definitive classification without subsequent clinical corroboration. Given that many older adults experience mixed pain syndromes with overlapping nociceptive and neuropathic features, 3 this binary stratification may overstate the role of neuropathy in frail subgroups. Clinicians using these results for therapeutic decisions, such as selecting anticonvulsants or serotonin-norepinephrine reuptake inhibitors, may risk overtreatment unless a confirmatory neurological examination is included.

The third concern relates to the analytical handling of psychological burden. The Geriatric Psychosocial Assessment of Pain-Induced Depression effectively captures pain-related emotional sequelae; however, its interpretation in the regression model lacks granularity. Frail participants had significantly higher depression scores; however, the study did not explore whether depressive symptoms mediate the relationship between pain and frailty. This distinction is not merely academic: if pain-induced depression drives functional decline, then interventions must prioritize mental health alongside analgesia. Without such modeling, the relative contribution of psychological distress remains unclear, limiting the translational impact of the study.

We commend the authors for assembling a richly-characterized cohort and applying validated multidimensional tools to dissect chronic pain in geriatric populations. Their findings underline the pressing need for frailty-aware pain assessments and multidisciplinary management strategies. Future studies using longitudinal and mediation-based models could clarify the causal directions and optimize care for this high-risk population.
